# Never Safe

**Published:** 1870-02

**Authors:** 


// HALL'S

JOURNAL OF HEALTH.

VOL. XVII. FEBRUARY, 1870. No. 2.

### NEVER SAFE.

IN 1835, a young man commenced business in New York City; he had but few friends, and they were as poor as himself; he was patient, industrious, economical, and temperate. There were no "lectures" in those days, nor Christian Associations to fold their protecting arms around the young men in a large city; the theater was always open, but when night came he went to bed. As he was at his place of business early and late, he received chance calls now and then from persons who could not wait, and as he was always ready for a job, and when he got one did it promptly and well, and at a very moderate charge, the persons thus served very naturally employed him again. In the course of a few years it was noticed that he could not be had on call as formerly; if persons wanted work, instead of doing it the same day, he would put it off sometimes for a week or more; but his customers felt so sure that he would come at the day, and the hour, and more, that he would do it well, they preferred waiting. One day a gentleman who had patronized him for a number of years, and had sent him several profitable customers, was not very well pleased at being put off for ten days, when it suited him so much better to have it done next day, and remonstrated with him, saying, "You used to come to my house on the instant, but now I have always to wait from two to ten days." The young man explained to him the reason that he had so much work to do that he could not find reliable hands enough to perform it; consequently, at certain seasons he could not avoid falling behindhand, and that the only just method was to serve his customers in turn; but even to do that according to promise, he and his men had sometimes to work all night; "but," said he, "I never fail to meet an engagement." He prospered. At fifty years of age he retired from business, with an ample fortune. Before he was sixty, in fact, in about five years, he died a degraded, drunken sot. Having nothing to do, time hung heavily. Being in perfect health he had a good appetite—indulged it, and sat around the house, lolled on the sofa, took "naps" in the day-time, and as a result did not sleep well at night; taking but little exercise and eating heartily, he soon began to suffer from indigestion, or dyspepsia; after each meal he would feel full, oppressed, uncomfortable, and to relieve himself, at the suggestion of a friend, he took a "sip of brandy;" he felt better; next day he took another; then a little more; soon he took it three times a day; then before meals, as well as after, with the result already detailed.

Thus it is, that this side heaven no man is safe from a drunkard's grave, except he who never tastes a drop of liquor. It might have been thought that fifty years of abstinence would have been a perfect guaranty against a vice so degrading, but it was not.

We can not refrain here from making a digression for the benefit of young men, and point out to them the way of securing a fortune in New York in any kind of useful business from making a shoe to building a locomotive. Go to your work at the hour appointed, do it well, and at a reasonable charge.

There he is in South America, at the age of sixty years, away from wife and children, and friends, and home, and he can never return. Forty-six years ago he was a bright, smooth-faced lad, with a large confiding eye and open countenance. He was an orphan, without a home, without occupation; he solicited and obtained the berth of sweeping out a bank every morning for a few cents a day. He was never behind time, and not only did his work well, but was always prompt to do any additional little thing which he was asked to do, without ever asking additional pay; if any money was found on the floor, behind or outside the counter, he was sure to hand it to the cashier, even if it were but a copper penny. In a little while he was employed as messenger to carry checks and other valuable papers, with an increased salary; but years passed before he was employed to carry money; bank men were careful in those days. The time did come, however, when he was intrusted with small sums, and by slow degrees it amounted to thousands at a time, and tens of thousands in a day. During these years he employed much of his spare time in acquiring a plain handwriting; every letter was made distinct and perfect by itself, almost like copperplate. He was made clerk of the bank records, then appointed to receive all the money, and finally was made cashier of one of the largest and best managed banks in New York. The whole business community reposed in him the most implicit confidence, and seldom has Wall Street been more astounded than it was one sunny morning, four months ago, by the announcement that the "cashier of the old and well-established and honorable bank of ———" had absconded, leaving a deficit of over a hundred thousand dollars, which meant that he had taken that much money belonging in part to helpless widows and orphan children, and that this thing had been going on for five or six years; that he had been deliberately for that long period committing a series of crimes worse and meaner than thieving. "Why," said a high bank officer a few days later, who had just returned from a visit to the wife and children of this man, to leave her money out of his own pocket to feed them from day to day, "if I had been away from home with him, and suddenly taken ill, and expected to die, and had a million of dollars in my pocket, I should not have hesitated an instant to place it in his hands to deliver to my wife." Surely no man is ever safe who could persuade himself to use one cent of money which belonged to another, without the owner's consent. This was the ruin of the cashier; he had the control of millions of money belonging to others who trusted wholly to him. At first he took a small amount to speculate on in Wall Street, as he saw so many getting rich around him. He lost. He took more and lost, with the result as above.

To be safe then from a drunkard's grave, never taste a drop of liquor. To be safe from a meaner fate than that of a thief, never use one cent of another's money without the explicit consent of the owner.

